# Supplementary material for: Neuroligin-mediated neurodevelopmental defects are induced by mitochondrial dysfunction and prevented by lutein in C. elegans
Source: Nat Commun. 2022 May 12;13:2620. doi: 10.1038/s41467-022-29972-4 (PMC9098500; doi:10.1038/s41467-022-29972-4)
Supplement: Supplementary file 1 — Supplementary Information [file 41467_2022_29972_MOESM1_ESM.pdf]

Supplementary Information

Table of Content

1. Supplementary Figures: 1 - 13
2. Supplementary Tables: 1 - 7
3. Supplementary Methods
4. Supplementary information references

Supplementary Figures

Supplementary Figure 1

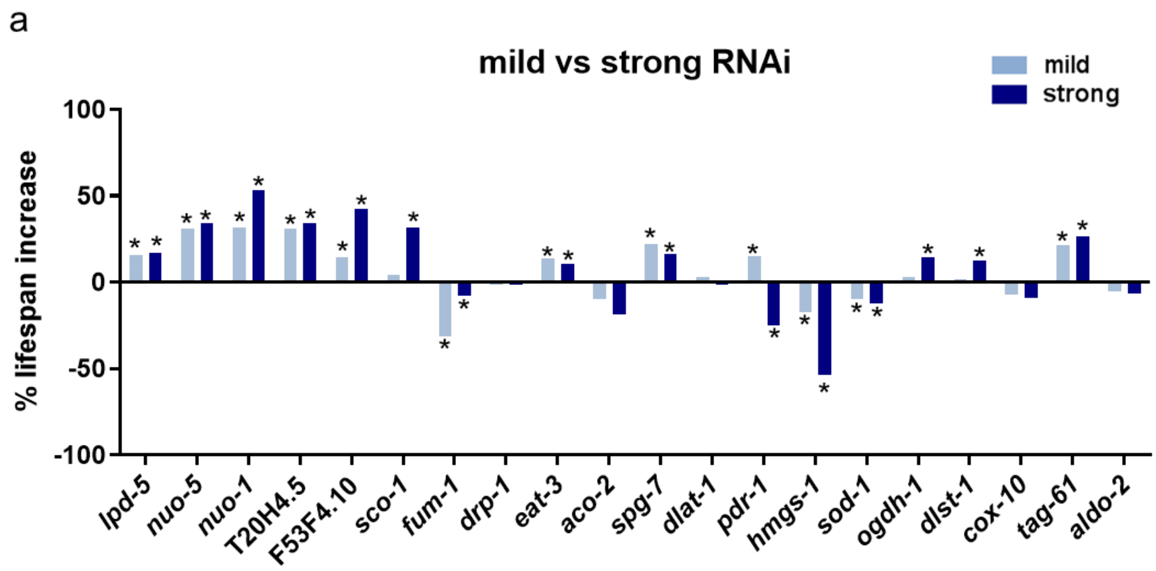

Supplementary Figure 1. Effect of RNAi against the mitochondrial proteins.
a) Summary of survival analysis: % changes in mean lifespan. Wild-type (N2) worms fed bacteria expressing either *lpd-5*, *nuo-5*, T20H4.5, F53F4.10, *nuo-1*, C01F1.2, *fum-1*, *drp-1*, *eat-3*, *aco-2*, *spg-7*, *pdr-1*, *sod-1*, F23B12.5, W02F12.5, *tag-61*, F01F1.12 or F25B4.6 dsRNA. Bar graphs represent % changes in means lifespan compared to N2 fed empty-vector (control)**.** Asterisks denote statistical significance compared to control. Log-rank test, *p-value <0.0001. See Supplementary Table 2 and Supplementary Dara 1 for summary statistics from Kaplan-Meier survival analysis.

Supplementary Figure 2

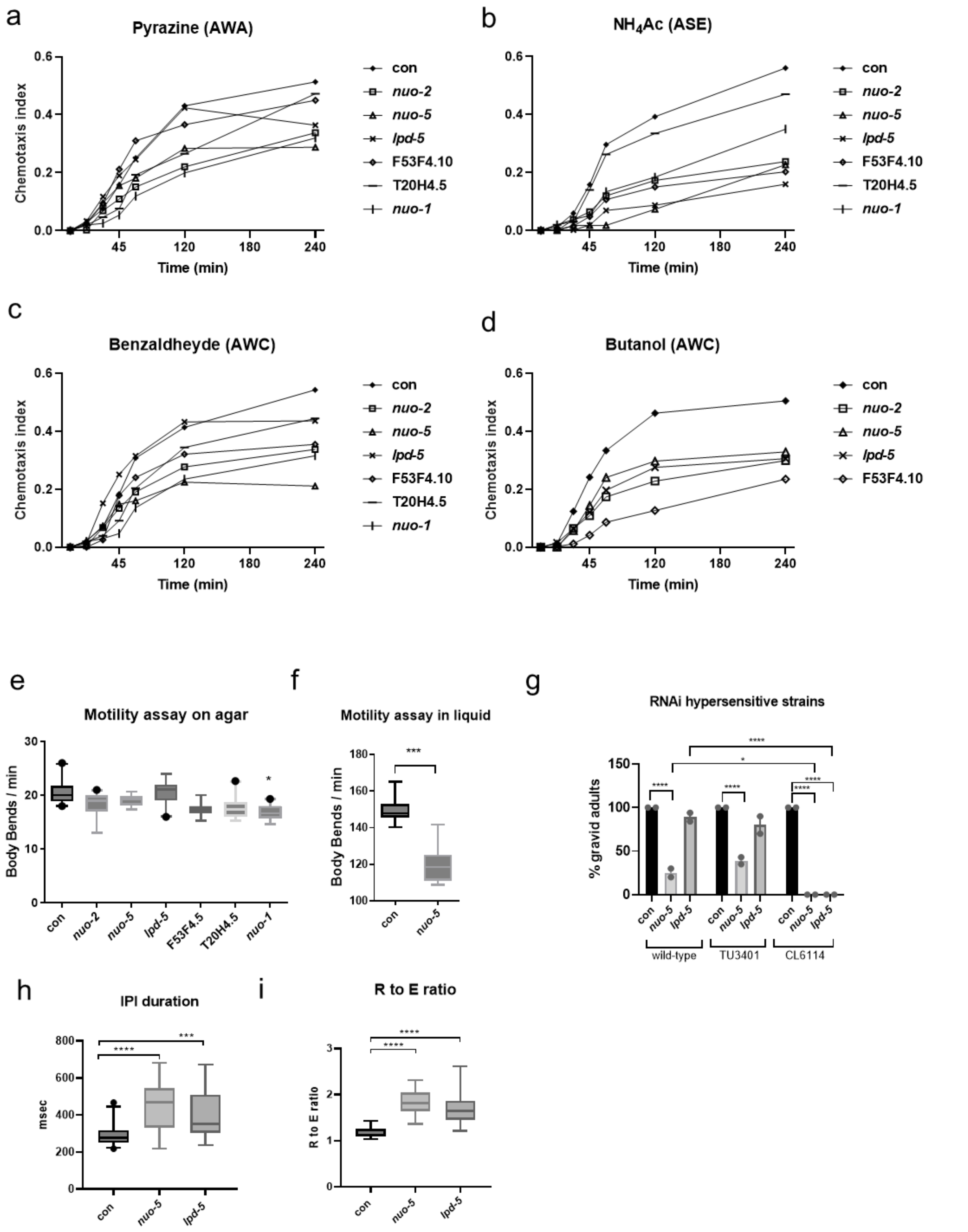

Supplementary Figure 2. Strong RNAi against the mitochondrial Complex I proteins affects chemotaxis, motility and pharyngeal pumping. a-d) impairment in sensory perception upon

strong RNAi against Complex I proteins. Chemotaxis curves obtained using 1µl of either 1% Pyrazine **(a),** 2.5M Sodium Acetate **(b),** 1% Benzaldehyde **(c)** or 1%Butanol **(d)** as attractant for the worms. Three biologically independent experiments, n≥=100. Two-way ANOVA followed by Tukey's multiple-comparisons test.

e) Motility assay on agar (one-way ANOVA) and **f)** in liquid media (two-tailed Student's t-test). Locomotion is quantified as body bend per minute. Three biologically independent experiments, n≥30. Box plots indicate median (middle line), 25th, 75th percentile (box) and 5th and 95th percentile (whiskers) as well as outliers (single points). p-values in **e**: con vs *nuo-1* p = 0.0491; p-values in **f**: con vs *nuo-5* p = 0.001. **g)** Comparison of development between N2 (wild-type) and RNAi sensitive *C. elegans* strain, specifically: TU3401: *sid-1*(pk3321); uls69[*myo2*p::mCherry + *unc-119*p::*sid-1*], a neuronal specific sensitive strain, and CL6114: *nre-1*(hd20) *lin-15b*(hd126), a strain with overall increased sensitivity to RNAi. Bars indicate the percentage of animals reaching the gravid adult (GA) stage (N2 and TU3401 reached the GA stage on control plates in three days, while CL6114 in four days). Bars represent means ± SEM and corresponding data points. Two biologically independent experiments, n=40. wild-type con vs wild-type *nuo-5* p < 0.0001, TU3401 con vs TU3401 *nuo-5* p < 0.0001, CL6114 con vs CL6114 *nuo-5* p < 0.0001**,** CL6114 con vs CL6114 *lpd-5* p < 0.0001, wild-type *nuo-5* vs CL6114 *nuo-5* p = 0.0403, wild-type *lpd-5* vs CL6114 *lpd-5* p < 0.0001**.** **h**) IPI (interpump interval) duration and **i**) Median R to E ratio were measured using the ScreenChipTM System (InVivo Biosystems). Box plots indicate median (middle line), 25th, 75th percentile (box) and 5th and 95th percentile (whiskers) as well as outliers (single points). Three biologically independent experiments, n≥30. p-values in **h:** con vs *nuo-5* p < 0.0001, con vs *lpd-5* p = 0.0004; in **i**: con vs *nuo-5* p < 0.0001, con vs *lpd-5* p < 0.0001. Raw data for all panels are provided as a Source Data file.

Supplementary Figure 3

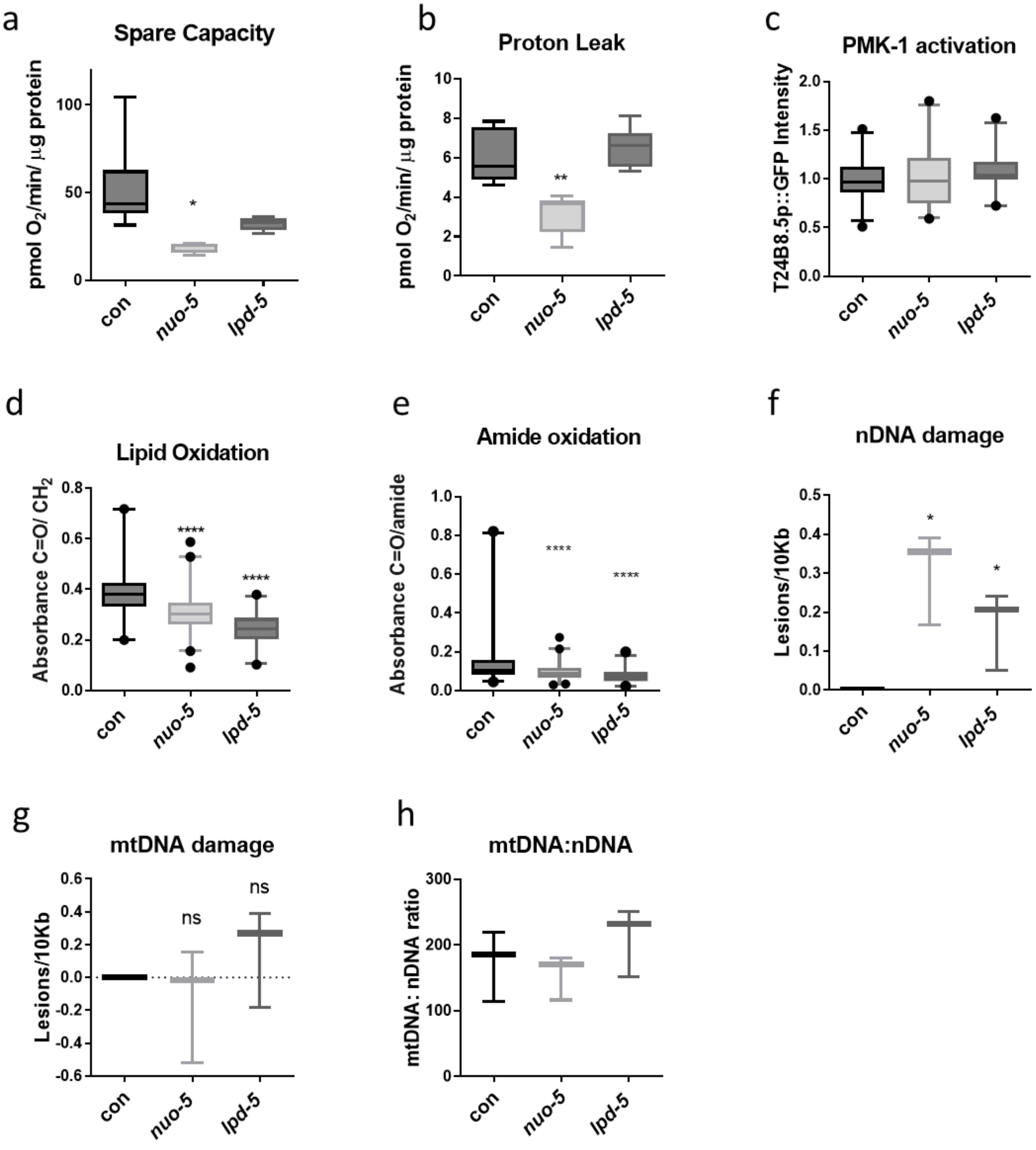

Supplementary Figure 3. Strong RNAi against the mitochondrial Complex I proteins does not lead to significant signs of oxidative damage. a) Proton leak and **b**) Spare capacity were measured using the Seahorse XF24 Analyzer. Three biologically independent experiments n≥1000 per group. p-values in **a**: con vs *nuo-5* p = 0.0012, in **b**: con vs *nuo-5* p = 0.0036. **c)** Quantification of GFP expression in a WT strains expressing the *agIs219* transgene, which is comprised of the promoter of a PMK-1-regulated gene, T24B8.5 fused to GFP and provides an *in vivo* sensor of

PMK-1 pathway activity. Two biologically independent experiments, n≥40. **d**) Lipid oxidation and **e**) amide oxidation assessed with SR-μFTIR. Three biologically independent experiments, n≥75 per group. p-values in **d:** con vs *nuo-5* $p < 0.0001$, con vs *lpd-5* $p < 0.0001$, p-values in **e**: con vs *nuo-5* $p < 0.0001$, con vs *lpd-5* $p < 0.0001$ **f)** Nuclear DNA (nDNA) damage, **g)** Mitochondrial DNA (mtDNA) damage and **h)** mtDNA copy number upon strong suppression of *lpd-5* and *nuo-5*. Three biologically independent experiments, n=18. p-values in **f:** con vs *nuo-5* $p = 0.0117$, con vs *lpd-5* $p = 0.0468$.

In all figure's panels worms were fed as in Fig 2 and statistical analysis was carried out using one-way ANOVA. Box plots indicate median (middle line), 25th, 75th percentile (box) and 5th and 95th percentile (whiskers) as well as outliers (single points).

Supplementary Figure 4

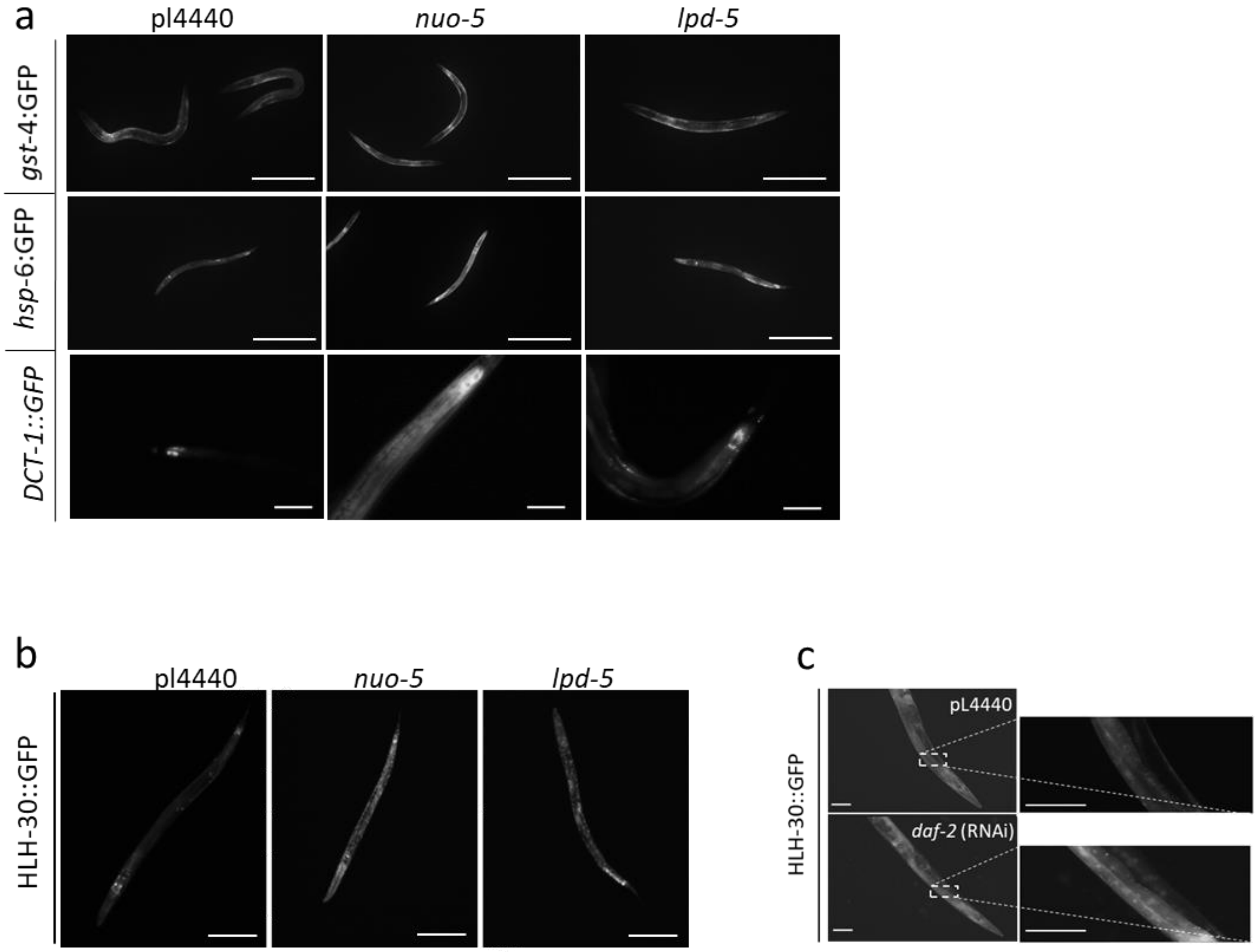

Supplementary Figure 4. Strong RNAi against the mitochondrial Complex I proteins differentially affect the expression of mitochondrial stress response genes. a) Representative pictures of GFP reporter strains upon *nuo-5* and *lpd-5* RNAi. Scale bar 370 µm for p_{gst-4}::GFP and p_{hsp-6}::GFP and 50 µm for p_{dct-1}DCT-*1*::GFP. Worms are L3 larvae. The expression was measured in four biologically independent experiments, n≥36, with similar results. **b)** Representative pictures of nuclear translocation of P_{hlh-30}::HLH-30::GFP upon *nuo-5 and lpd-5* RNAi. Worms are L3 larvae. Scale bars 100 µm. Expression was measured in four biologically independent experiments, n≥56 **c**) Representative pictures of nuclear translocation of P_{hlh-30}::HLH-30::GFP upon *daf-2* RNAi in one day old worms. Scale bars 100 µm. Three biologically independent experiments with similar results.

Supplementary Figure 5

a

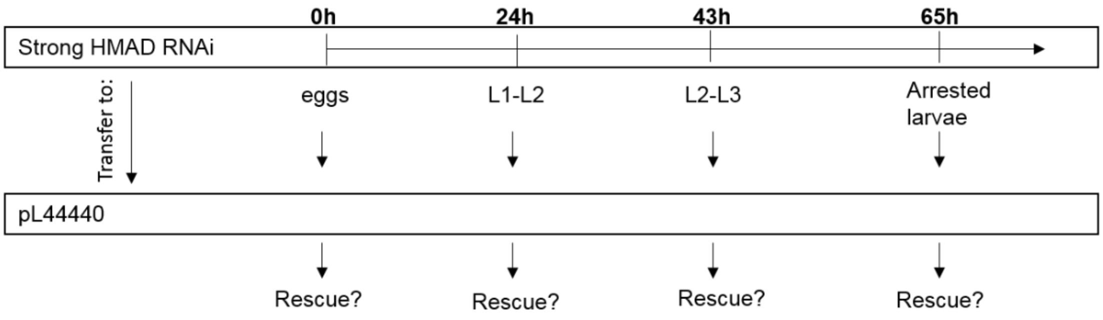

RNAi feeding	Always on pL4440	Moved on pl4440 as eggs	Moved on pl4440 as L1-L2	Moved on pl4440 as L2-L3	Moved on pl4440 after 65h
Development stage and fertility (4 days after hatching)		Gravid adult less eggs than control	Some gravid adult few eggs	No Gravid adult and no eggs	Arrested larvae
	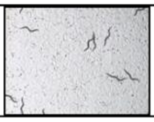	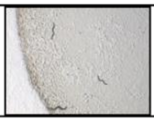	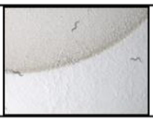	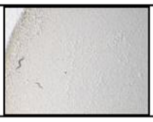	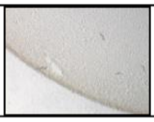

b

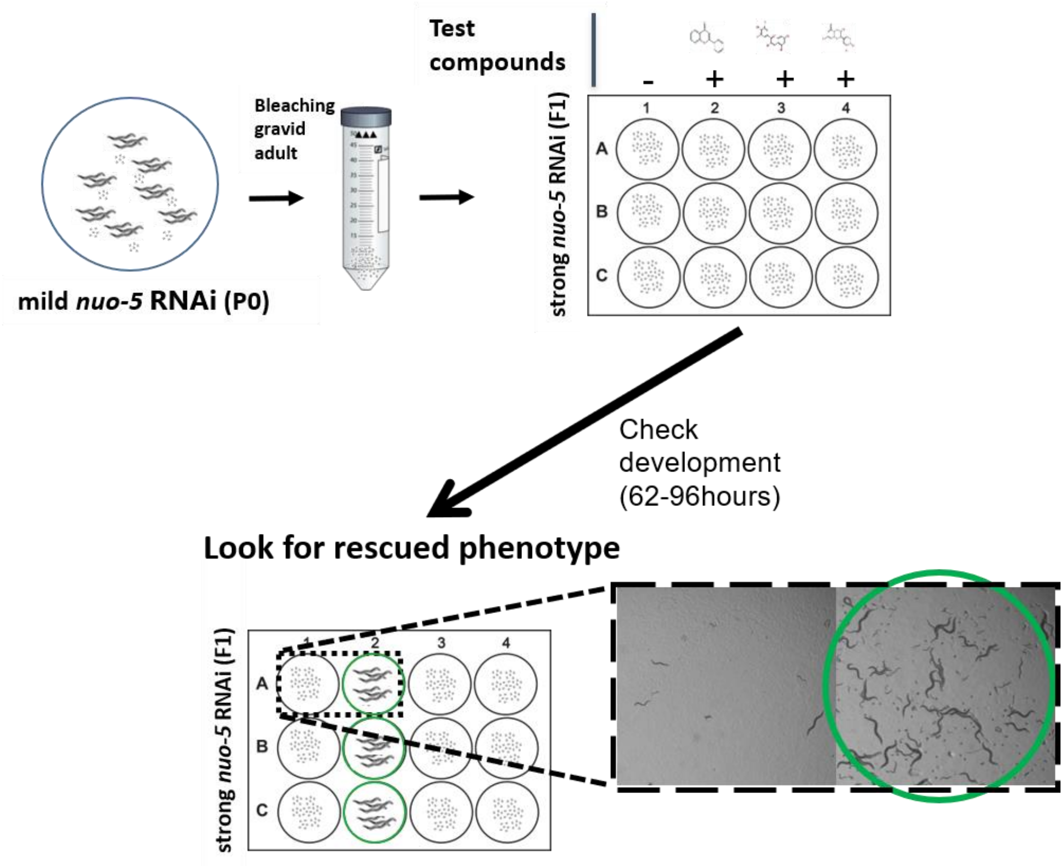

Supplementary Figure 5. Drug suppressor screening proof of principle and workflow. a) Schematic experimental strategy to proof the reversibility of the strong phenotype. Animals were cultured on RNAi plates (strong treatment); then worms were moved on control plates (fed the empty vector pL4440) either as eggs or at different larval stages. The development was checked for rescue on day 4 after egg hatching (representative pictures of a partial rescue obtained returning the nematodes on pL4440 within 24 hours). **b**) Flowchart with representative pictures of the compounds screen in search of suppressors of the *nuo-5* and *lpd-5* strong (developmental arrest) phenotype.

Supplementary Figure 6

a

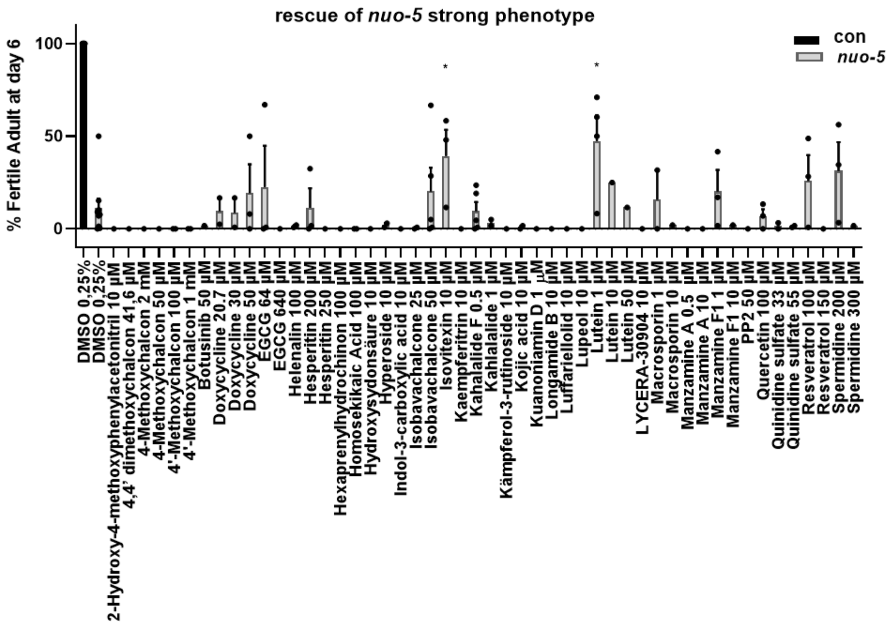

b

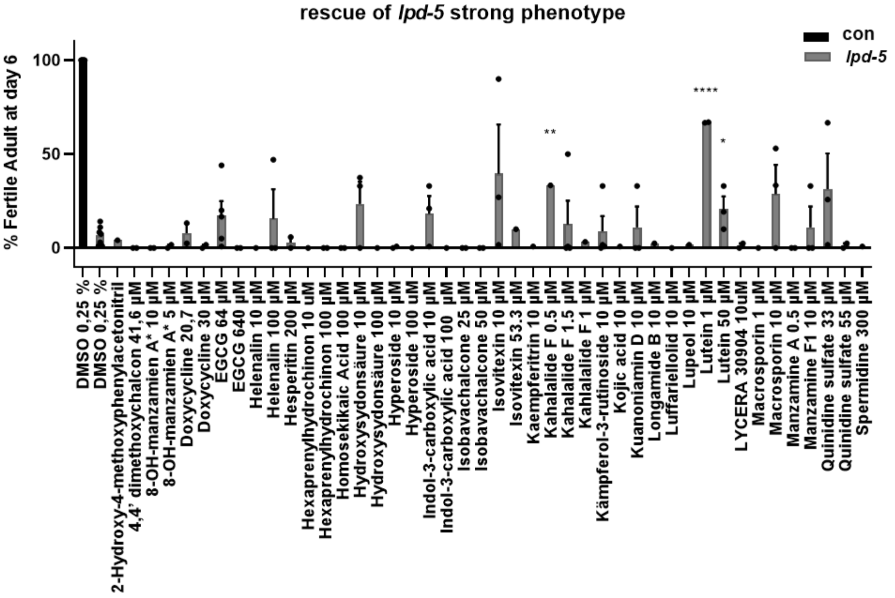

Supplementary Figure 6. Results of suppressor screening. Bars show the percentage of animals which developed to gravid adult after 6 days from eggs ± SEM with corresponding data points. p-values in **a**: *nuo-5* DMSO vs *nuo-5* isovitexin 10 µM p = 0.0379, *nuo-5* DMSO vs lutein 1 µM p = 0.0102; p-values in **b:** *lpd-5* DMSO vs kahalalide F 0.5 µM p = 0.001, *lpd-5* DMSO vs lutein 1 µM p < 0.0001, *lpd-5* DMSO vs lutein 50 µM p = 0.022. n≥40, one-way ANOVA. Data for all panels are provided as a Source Data file.

Supplementary Figure 7

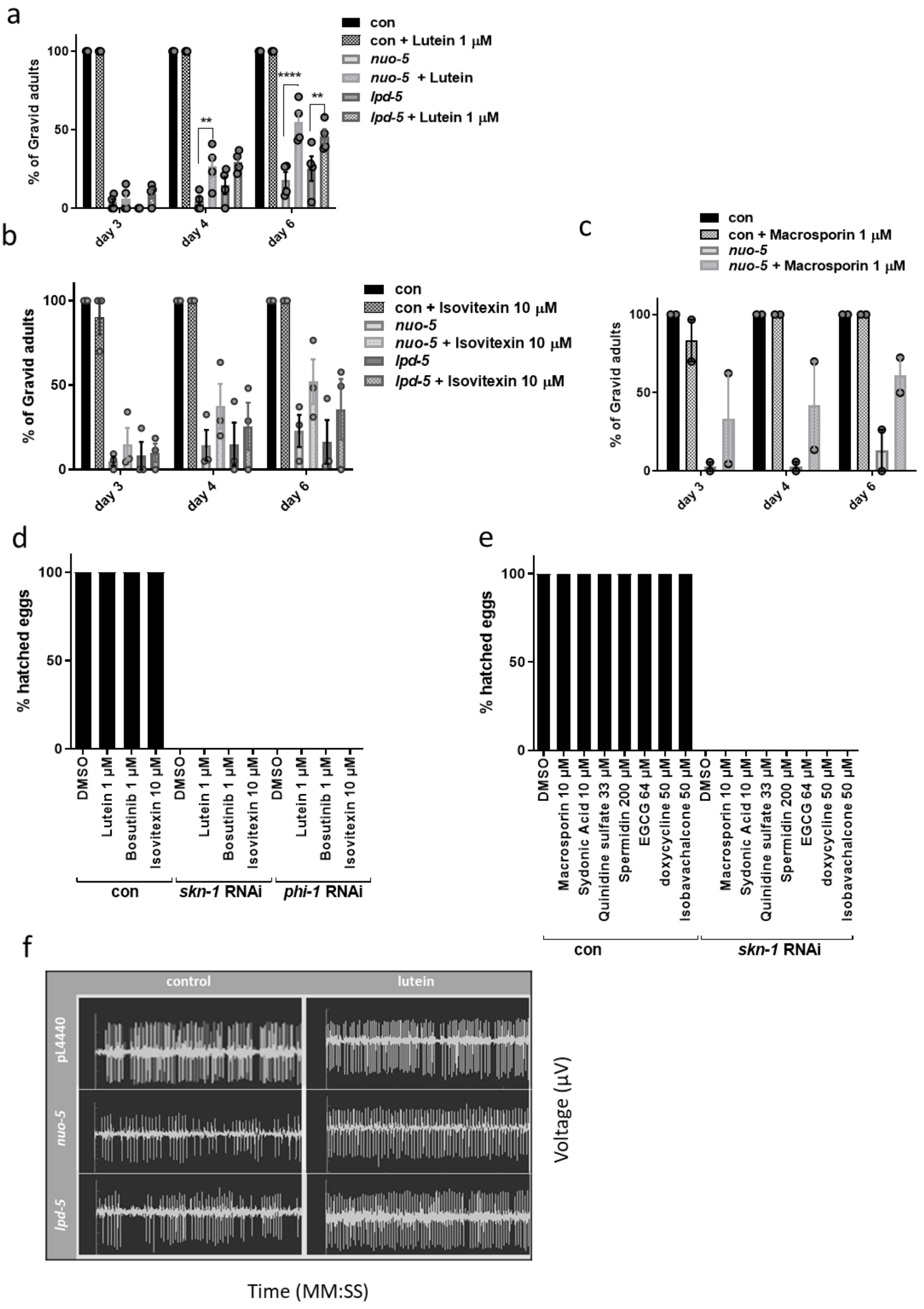

Supplementary Figure 7. Effects of selected drugs on developmental rate and pharyngeal pumping.

a-c) Development of *nuo-5* and *lpd-5*-depleted worms treated with either 1µM lutein or DMSO (**a**), with 1µM isovitexin or DMSO **(b**), or with 1µM macrosporin or DMSO (**c**). Two-way ANOVA. p-values in **a:** for day 4, *nuo-5* vs *nuo-5* + lutein p = 0.0028, for day 6, *nuo-5* vs *nuo-5* + lutein p < 0.0001, *lpd-5* vs *lpd-5* + lutein p = 0.0063. Bar graphs represent means ± SEM and corresponding data points. Four (**a**), three (**b**) and two (**c**) biologically independent experiments, n≥30.

d-e) The typical embryonic lethality induced *phi-7* (**d-e**) and *skn-1* (**e**) RNAi is conserved upon drugs treatment. Bar graphs represent percentage of animals reaching adulthood. Two biologically independent experiments, n≥40.

f) Representative EPGs, 20 seconds frame of ~2 minutes recordings are shown for each condition. Pharyngeal pump frequency was measured using the ScreenChipTM System (InVivo Biosystems). Three independent experiments with similar results, n=45. Data for all panels are provided as a Source Data file.

Supplementary Figure 8

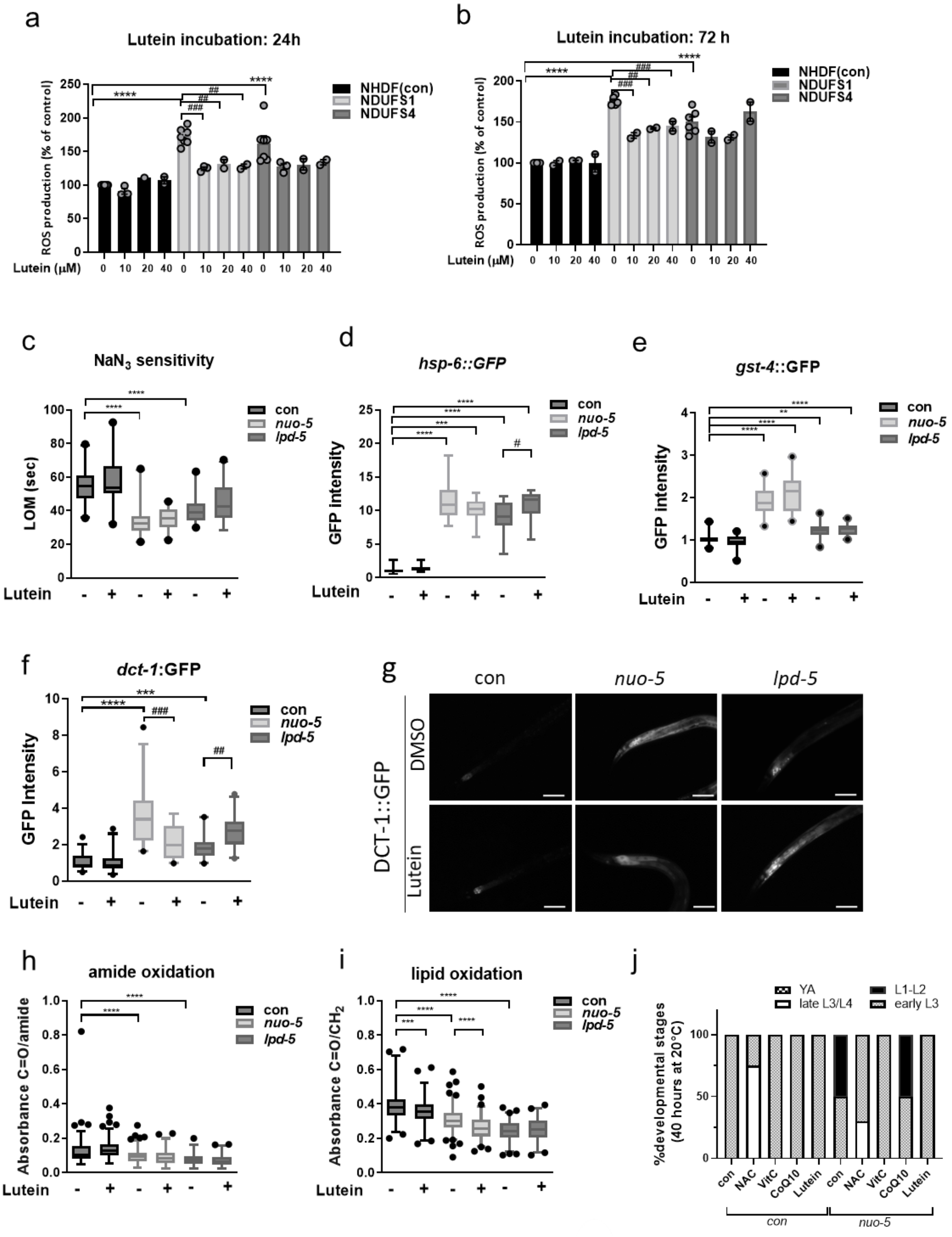

Supplementary Figure 8. Effects of lutein on different parameters in *nuo-5* and *lpd-5 C. elegans* mutants and patients' derived cells with mutations in the corresponding genes. a) ROS production in human skin fibroblasts with lutein 24 hours incubation and **b**) 72 hours incubation. Different concentrations of lutein were used. Three biologically independent experiments**.** Bar graphs represent means ± SEM with corresponding data points. p-values in **a:** con vs NDUFS1 p < 0.0001, NDUFS1 vs NDUFS1 + lutein 10 µM p = 0.0002, NDUFS1 vs NDUFS1 + lutein 20 µM p = 0.0036, NDUFS1 vs NDUFS1 + lutein 40 µM p = 0.0019, con vs NDUFS4 p < 0.0001; in **b:** con vs NDUFS1 p < 0.0001, NDUFS1 vs NDUFS1 + lutein 10 µM p = 0.0002, NDUFS1 vs NDUFS1 + lutein 20 µM p = 0.0001, NDUFS1 vs NDUFS1 + lutein 40 µM p = 0.0006, con vs NDUFS4 p < 0.0001. One-way ANOVA. **c**) Sensitivity to 10 mM sodium azide, LOM = last observed movement. con vs *nuo-5* p < 0.0001, con vs *lpd-5* p < 0.0001. **d**) Quantification of GFP expression in WT strains expressing the p_{hsp-6}*::GFP*. con vs *nuo-5* p < 0.0001, con vs *lpd-5* p < 0.0001, con vs *nuo-5* + lutein p < 0.0001, *lpd-5* vs *lpd-5* + lutein p = 0.0432, con vs *lpd-5* + lutein p < 0.0001. **e)** Quantification of GFP expression in WT strains expressing the p_{gst-4}*::GFP*. con vs *nuo-5* p < 0.0001, con vs *lpd-5* p = 0.0219, con vs *nuo-5* + lutein p < 0.0001, con vs *lpd-5* + lutein p < 0.0001. **f**) Quantification of GFP expression in WT strains expressing the p_{dct-1}::DCT-1::GFP transgene and corresponding representative pictures (**g**) Scale bar 50 µm**.** Two biologically independent experiments, n≥36. Box plots indicate median (middle line), 25th, 75th percentile (box) and 5th and 95th percentile (whiskers) as well as outliers (single points). con vs *nuo-5* p < 0.0001, con vs *lpd-5* p = 0.0018, *nuo-5* vs *nuo-5* + lutein p = 0.0005, *lpd-5* vs *lpd-5* + lutein p = 0.0048. Animals are fed as in **Fig. 2** with and without lutein (1 µM final concentration) administration. Asterisks (*) denote significant differences vs control, # denote differences among conditions. **h**) Amide oxidation and **i**) lipid oxidation, both assessed with SR-µFTIR. Three biologically independent experiments, n≥45 per group Box plots indicate median (middle line), 25th, 75th percentile (box) and 2.5th and 97.5th percentile (whiskers) as well as outliers (single points). p-values in **h**: con vs *nuo-5* p < 0.0001, con vs *lpd-5* p < 0.0001; p-values in **i:** con vs con + lutein p = 0.0005, con vs *nuo-5* p < 0.0001, *nuo-5* vs *nuo-5* + lutein p < 0.0001, con vs *lpd-5* p < 0.0001. **j)** Comparison of development between control and worms fed *nuo-5* RNAi treated either with DMSO or the indicated antioxidant. Bars indicate the percentage of different developmental stages after 40 hours from egg lay. L1 -L2, 1st to 2nd larval stage; early L3, beginning of 3rd larval stage; late L3/L4, end of 3rd larval stage and beginning of 4th larval stage. Two biologically independent experiments, n=40. Two-way ANOVA unless otherwise specified. Data for all panels are provided as a Source Data file.

Supplementary Figure 9

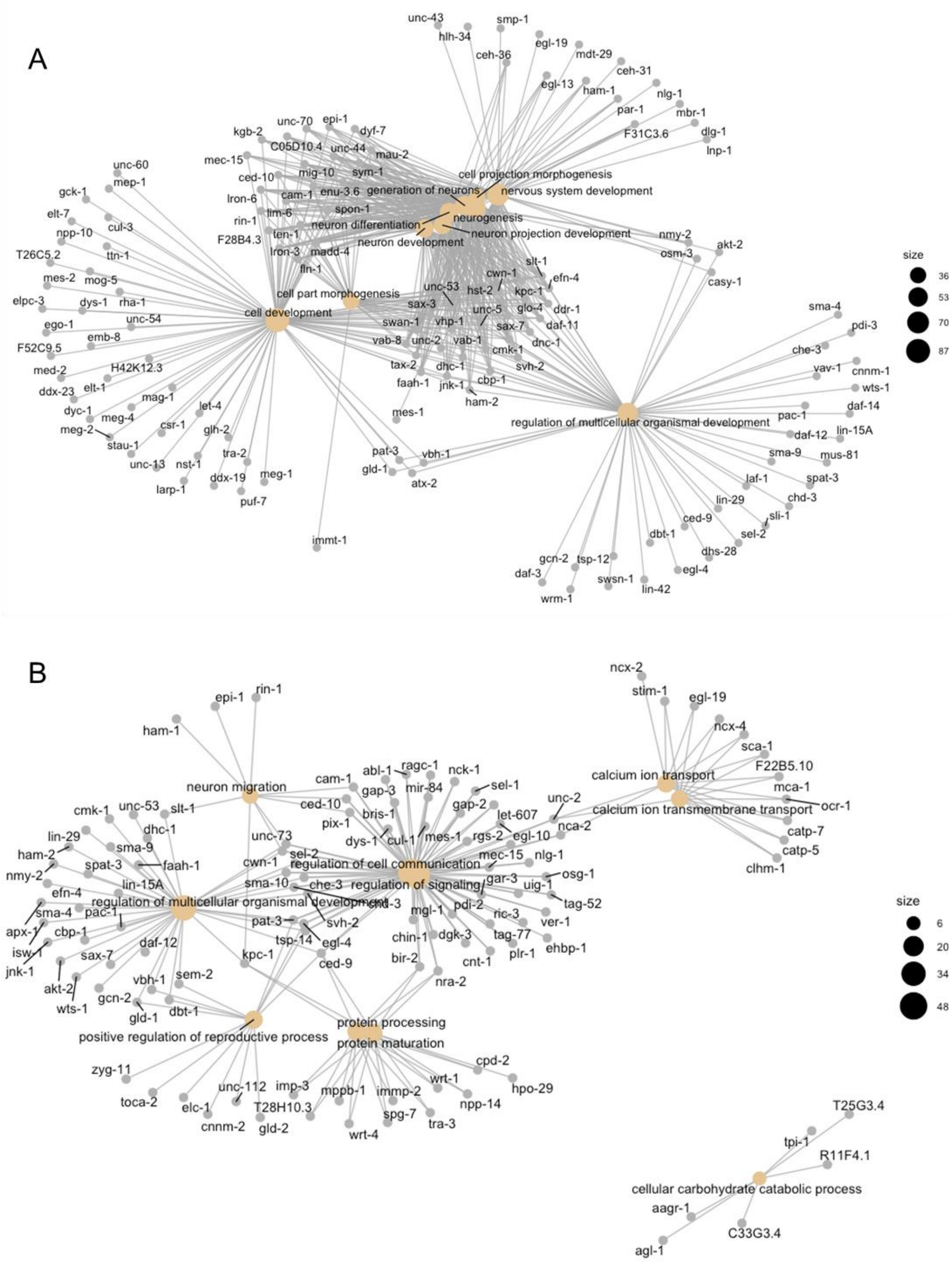

Supplementary Figure 9. Biological complexity network. a) Microarray analysis comparing control vs *nuo-5* strong treated animals (results showed in **Fig. 5**); **b**) control vs *nuo-5* strong + lutein. Created in R, using Bioconductor.

Supplementary Figure 10

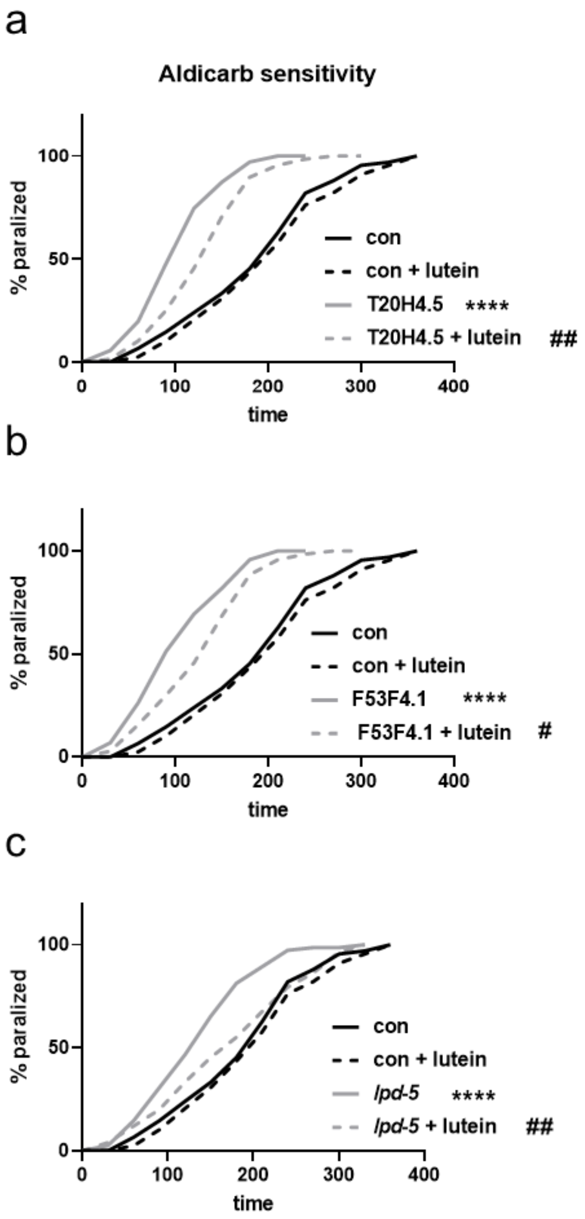

Supplementary Figure 10. Aldicarb sensitivity and lutein effect in other CI clones. Aldicarb-induced paralysis curves on plates containing 0.5 mM aldicarb of worms fed bacteria transformed either with empty-vector (con, black lines) or with vector expressing dsRNA against **a**) T20H4.5 or **b**) F53F4.1 or **c**) *lpd-5* (light grey lines) and left untreated or treated with 1µM Lutein. Log-rank test, p-values in **a**: con vs TH204.5 $p < 0.0001$, TH204.5 vs TH204.5 + lutein $p = 0.0004$; p-values in **b**: con vs F53F4.10 $p < 0.0001$, F53F4.10 vs F53F4.10 + lutein $p = 0.0037$; p-values in **c:** con vs *lpd-5* $p < 0.0001$, *lpd-5* vs *lpd-5* + lutein $p = 0.0005$. Asterisks (*) denote significant differences vs control, # denote differences among conditions. Three biologically independent experiments. n≥75. Data for all panels are provided as a Source Data file.

Supplementary Figure 11

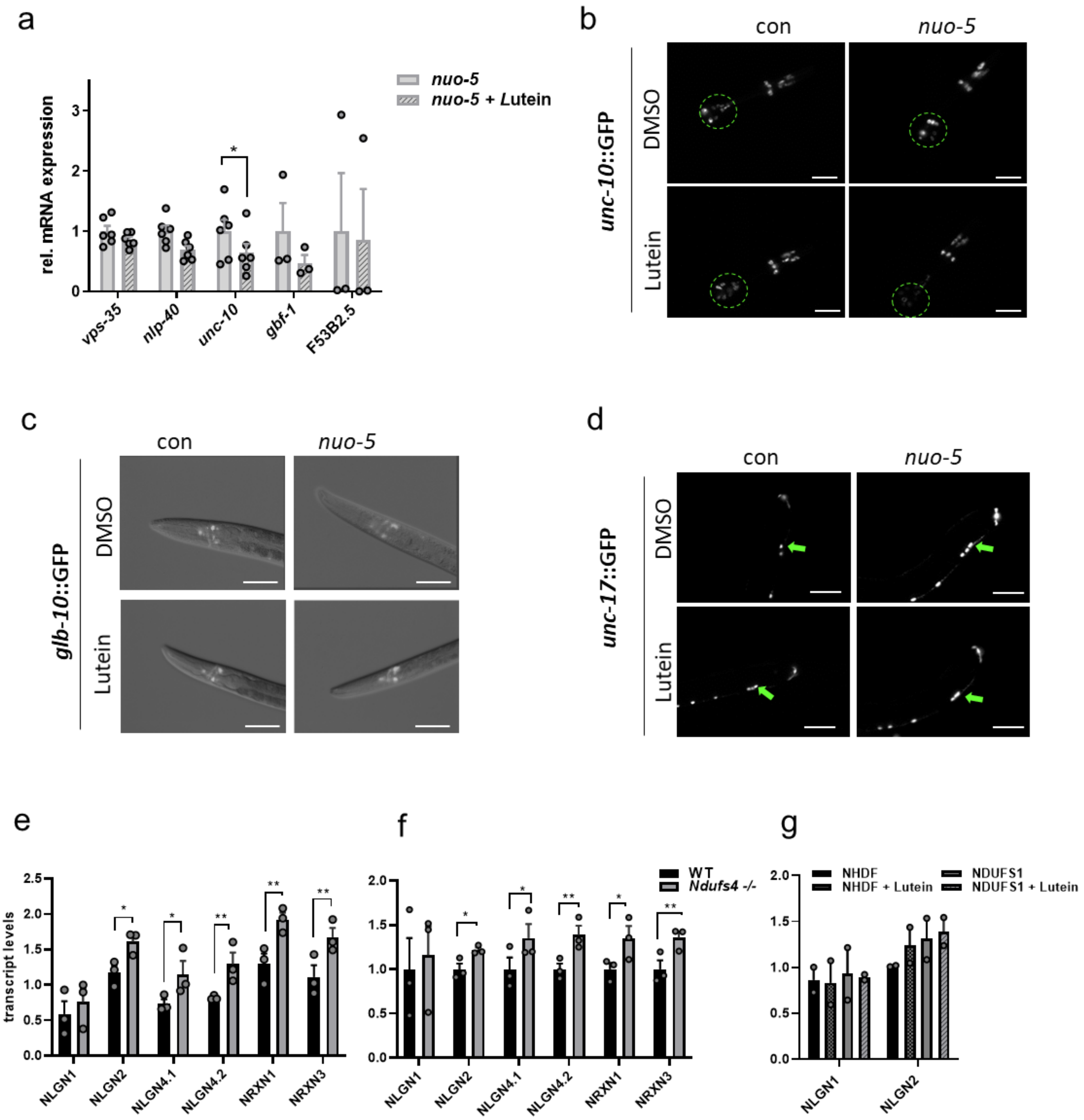

Supplementary Figure 11. Lutein affects synaptic genes' expression.

a) Relative mRNA expression of *vps-35, nlp-40, unc-10, gbf-1* and F53B2.5 assessed by qPCR. p-value for *unc-10* p = 0.0392.

b) Representative pictures of p$_{unc\text{-}10:}$:GFP in head neurons (dashed green circles show posterior pharyngeal bulbs, where the GFP expression mostly changes). Scale bar 20 µm. **c)** Representative pictures of p$_{glb\text{-}10}$::GFP in head neurons. Scale bar 50 µm. Three biologically independent experiment with similar results. **d**) Representative pictures of p$_{unc\text{-}17}$::GFP in head neurons (green arrows indicate the neuron considered for quantification). Scale bar 50 µm. **e-f)** Expression levels of neuroligin (NLGN1-2 and 4) and neurexin (NRX1 and 3) transcripts in brains of Ndufs4$^{-/-}$ mice compared to healthy animals, normalized to **e**) GAPDH or **f**) HPRT**,** assessed by qPCR. Three biologically independent experiments, n=3. p-values in **e:** NLGN2 p = 0.0155, NLGN4.1 p = 0.0135, NLGN4.2 p = 0.0049, NRXN1 p = 0.0025, NRXN3 p = 0.0025; p-values in **f**: NLGN2 p = 0.0183, NLGN4.1 p = 0.0247, NLGN4.2 p = 0.0082, NRXN1 p = 0.0011, NRXN3 p = 0.0027. **g**) NLGN1-2 transcripts in human fibroblasts, assessed by qPCR. 10 µM Lutein was administrated for 72 h. Two biologically independent experiments. Bar graphs represent means ± SEM and corresponding data points. One-way ANOVA. Raw data for all panels are provided as a Source Data file.

Supplementary Figure 12

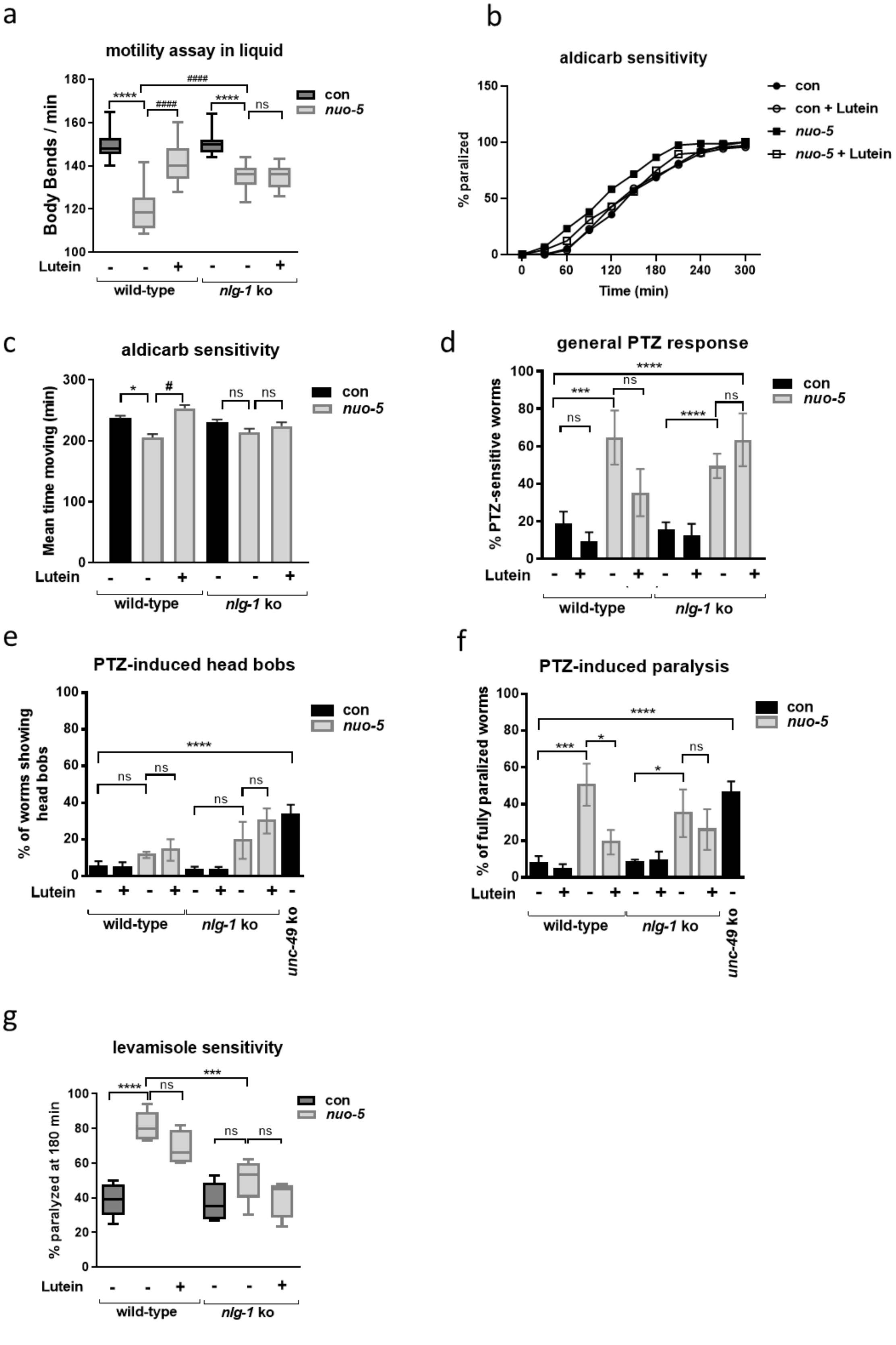

Supplementary Figure 12. Lutein effects are mediated by *nlg-1* suppression. a) Frequency of lateral swimming ("thrashing") movements quantified manually in liquid media for one minute. Box plots indicate median (middle line), 25th, 75th percentile (box) and 2.5th and 97.5th percentile (whiskers) as well as outliers (single points). Three biologically independent experiments, n=45. wild-type con vs wild-type *nuo-5* p < 0.0001, wild type *nuo-5* vs wild-type *nuo-5* + lutein p < 0.0001, wild-type *nuo-5* vs *nlg-1 ko nuo-5* p < 0.0001, *nlg-1* ko con vs *nlg-1 nuo-5* + lutein p < 0.0001. **b**) Aldicarb-induced paralysis curves and **c**) mean time moving on aldicarb plates of control and *nuo-5* in the presence or absence of lutein. (Aldicarb concentration: 0.5 mM). Three biologically independent experiments, n=≥75 per group. wild-type con vs wild-type *nuo-5* p < 0.022, wild type *nuo-5* vs wild-type *nuo-5* + lutein 0.011. **d)** PTZ general response, comprehensive of **e**) PTZ-induced head bobs. Four biologically independent experiments, n=60. Bars indicate the percentage of animals showing head convulsions (head bobs) phenotypes after 1 hour of exposure to 2.5 mg/ml PTZ and **f**) PTZ induced full body paralysis. Four biologically independent experiments, n=15-17. Bars indicate the percentage of animals showing full body paralysis after 1 hour of exposure to 2.5 mg/ml PTZ. The *unc-49* knockout strain was used as positive control. Bar graphs represent means ± SEM. p-values in **d:** wild-type con vs wild-type *nuo-5* p = 0.0010, *nlg-1* ko con vs *nlg-1 nuo-5* p < 0.0001, wild-type vs *unc-49* ko p < 0.0001; in **e:** wild-type vs *unc-49* ko p < 0.0001; in **f:** wild-type con vs wild-type *nuo-5* p = 0.0010, wild type *nuo-5* vs wild-type *nuo-5* + lutein p = 0.0199, *nlg-1* ko con vs *nlg-1* ko *nuo-5* p = 0.042, wild-type vs *unc-49* ko p < 0.0001. **g**) Sensitivity to Levamisole shown as mean survival on plates containing 50 μM Levamisole. Box plots indicate median (middle line), 25th, 75th percentile (box) and 2.5th and 97.5th percentile (whiskers) as well as outliers (single points). Four biologically independent experiments, n≥60. wild-type con vs wild-type *nuo-5* p < 0.0001, wild-type *nuo-5* vs *nlg-1* ko *nuo-5* p = 0.0019.

Two-way ANOVA, asterisks (*) denote significant differences vs control, # denote differences among conditions. Raw data for all panels are provided as a Source Data file.

Supplementary Figure 13

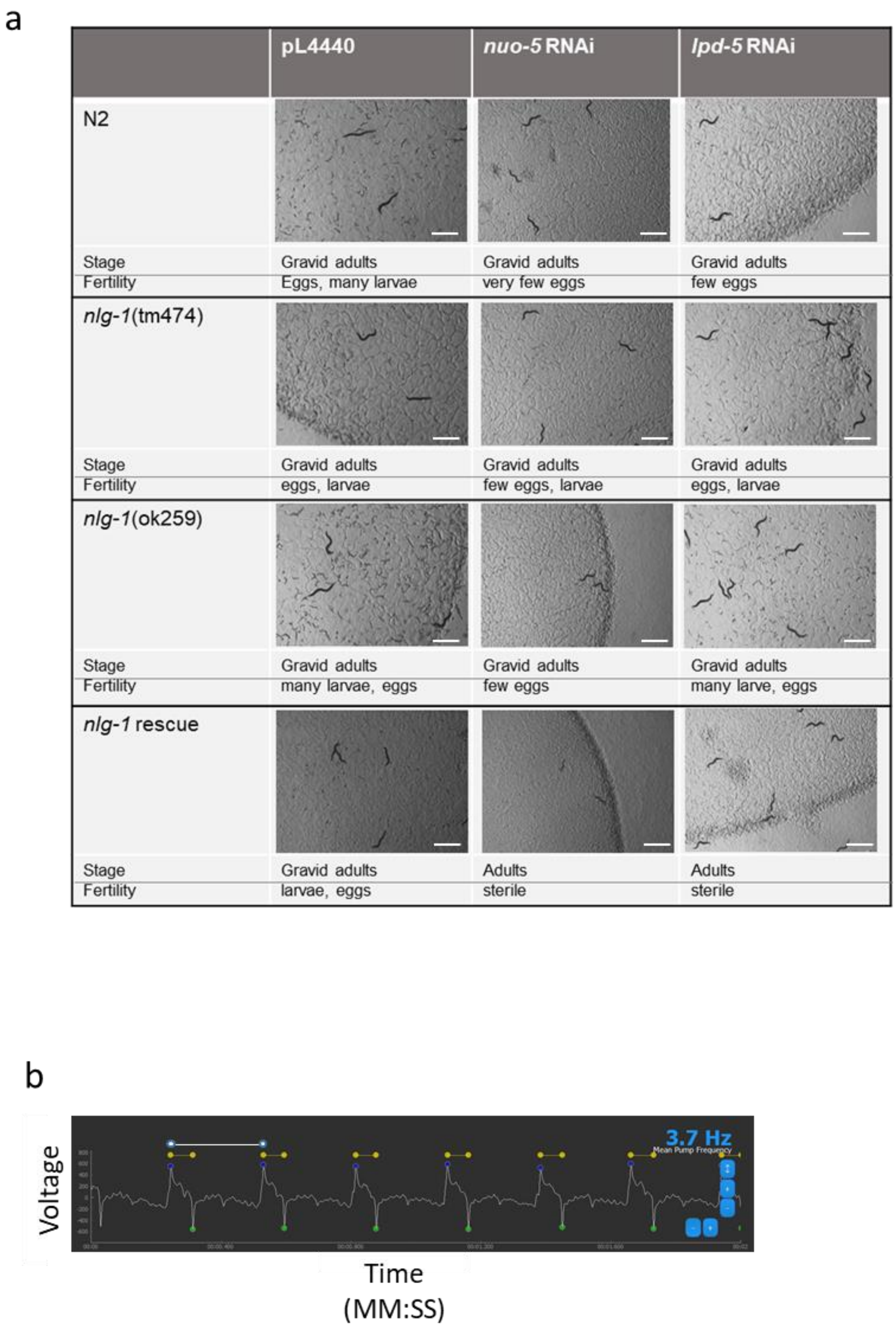

Supplementary Figure 13. Effects of gene silencing on development of RNAi hypersensitive strains and EPG representative track.

a) Representative pictures and brief developmental description of different C. *elegans* strains treated with mild RNAi against *nuo-5* or *lpd-5* (parental generation), after 4 days from egg-lay. *nlg-1* rescue corresponds to CRR104 transgenic strain (crrEx4 [pPD95.77 (*Pnlg-1*::*nlg-1*); pDD04NeoR (P*myo*-2::GFP)]. Pictures were acquired with a Leica MZ10 F modular stereo microscope connected to a color digital camera. Scale bars 1 mm. Three biologically independent experiments with similar results.
b) Representative EPG recording of a 5-days old worm. The voltage change is plotted against the time. Upon contraction of the pharynx, a positive voltage change can be observed as an excitatory spike (E spike, shown as blue dots). The relaxation of the pharynx muscle results in an adverse voltage change, the relaxation spike (R spike, shown as green dots). The horizontal yellow line represents the pump duration while the white line shows an inter-pump interval (IPI), the interval between one E spike and the next neighboring E spike. The R/E ratio is the ratio of the repolarization and depolarization waves in each pump (R and E waves) and is a parameter that indicates probable alterations in the normal muscle contraction/relaxation.

Supplementary Tables

Supplementary Table 1. List of genes whose different degree of silencing did not lead to consistent change in the observed phenotypes

Cosmid ID	Gene	Human ortholog, short gene description	Disease
F08A8.1	F08A8.1	ACOX-1, Acyl-CoA oxidase, complex IV	Pseudo-natal adrenoleukodystrophy
B0432.2	*djr-1.1*	PARK7	Parkinson's disease
T24H7.1	*phb-2*	PHB2 (prohibitin 2). Is involved in several processes, including defecation; gamete generation; and mitochondrion morphogenesis	
EEED8.9	*pink-1*	PINK1 (PTEN-induced kinase)	Parkinson' disease
ZK1248.14	*fzo-1*	Mfn-2, mitofusin1-GTPase/	Charcot Marie Tooth (CMT2)
F41G3.4	*fis-1*	fis-1, fission1, collaborate with DRP-1(dinamin related protein) in the mitochodniral fisison process	optic dystrophy
T05H10.6	*pdha-1*	PDHA1 (pyruvate dehydrogenase E1 subunit alpha 1) and PDHA2 (pyruvate dehydrogenase E1 subunit alpha 2)	pyruvate decarboxylase deficiency
F42A8.5	*sdhb-1*	SDHB, tag-55, Fe-S complex II	extradrenal pheocromocitoma
Y57A10A.15	Y57A10A.15	POLG, mtDNA pol	PEO-progressive exteranl ophtalmoplegia
T24C4.1	*ucr-2.3*	UQCRC2 (ubiquinol-cytochrome c reductase core protein 2). Predicted to have metalloendopeptidase activity	mitochondrial complex III deficiency nuclear type 5
R10E4.5	*nth-1*	NTHL1 (nth like DNA glycosylase 1)	familial adenomatous polyposis 3
M03C11.5	*ymel-1*	YME1L1 (YME1 like 1 ATPase)	optic atrophy 11
F45E4.9	*hmg-5*	TFAM (transcription factor A, mitochondrial). Is involved in mitochondrial DNA metabolic process.	
T01B11.4	*tag-316*	SLC25A4 (solute carrier family 25 member 4) and SLC25A5 (solute carrier family 25 member 5)	
Y43C5A.5	*thk-1*	TK1 (thymidine kinase 1). Is predicted to have thymidine kinase activity	
Y38F2AR.7	*ppgn-1*	SPG7 (SPG7 matrix AAA peptidase subunit, paraplegin)	hereditary spastic paraplegia 7
Y22D7AL.5	*hsp-6*	HSPA9 (heat shock protein family A (Hsp70) member 9)	autosomal dominant sideroblastic anemia 4
F46E10.8	*ubh-1*	UCHL1 (ubiquitin C-terminal hydrolase L1) and UCHL3 (ubiquitin C-terminal hydrolase L3)	
F13B9.8	*fis-2*	FIS1 (fission, mitochondrial 1). Is involved in apoptotic process	
C03G5.1	*sdha-1*	SDHA (succinate dehydrogenase complex flavoprotein subunit A)	dilated cardiomyopathy 1GG; mitochondrial complex II deficiency; and paraganglioma
F21G4.6	F21G4.6	HTT (huntingtin)	Huntington's disease

Supplementary Table 2. Lifespan summary statistics undiluted and diluted

Gene/ RNAi clone	RNAi power	Mean Lifespan ± SE (Days)	Lifespan change (%)[a]	P value vs control[b]	P value mild vs strong[c]	Sample size/ n trials
con		18,61 ± 0,26				330/4
spg-7	mild	22,79 ± 0,34	+22,4 [1]	< 0,0001		230/3
	strong	21,69 ± 0,32	+16,5	< 0,0001	0,023	230/3
con		18,13 ± 0,27				250/3
nuo-1	mild	23,91 ± 0,51	+31,8	< 0,0001		200/3
	strong	27,83 ± 0,68	+53,5	< 0,0001	< 0,0001	200/3
T20H4.5	mild	18,74 ± 0,32	+3,36	0,63		160/2
	strong	27,34 ± 0,66	+50,79	< 0,0001	< 0,0001	140/2
dlst-1	mild	18,49 ± 0,29	+1,98	2,13		200/3
	strong	20,37 ± 048	+12,34	< 0,0001	0,001	200/3
con	mild	17,83 ± 0,56				250/3
pdr-1	mild (1:10)	17,75 ± 0,27	- 0,44	5,32		200/3
	mild (1:50)	20,56 ± 0,47	+15,31 [1]	< 0,0001	< 0,0001	80/1
	strong	13,38 ± 0,22	-24,95	< 0,0001	< 0,0001	200/3
dlat-1	mild	18,41± 0,26	+3,25 [1]	0,2236		200/3
	strong	17,59 ± 0,30	-1,34	0,76	0,19	200/3
con		17,70 ± 0,28				235/3
tag-61	mild	21,54 ± 0,36	+21,46 [1]	< 0,0001		250/3
	strong	22,40± 0,41	+ 26,55	< 0,0001	0,73	220/3
con		17,55 ± 0,34				190/3
hmgs-1	mild (1:10)	11,32 ± 0,45	-35,49 [1]	< 0,0001		140/2
	mild (1:50)	14,54 ± 0,95	-17,15	< 0,0001	< 0,0001	80/1
	strong	8,07 ± 0,25	-54,0	< 0,0001	< 0,0001	140/2

All these clones showed a strong phenotype already in the parental generation (P0, see Fig. 1) when used undiluted, and the mild effect was thus achieved by diluting the dsRNA expressing bacteria (P0, 1/10 or 1/50).

[a] % increase normalized mean lifespan compared to control; [b] Kaplan-Meier survival analysis, Log-rank test against control; [c] Kaplan-Meier survival analysis, Log-rank test between mild and strong treatment.

Supplementary Table 3. Summary of chemotaxis assays in *C. elegans* models of Complex I deficiency

RNAi clone	RNAi power	NH_4Ac 2,5M (ASE)	Pyrazine (AWA)	Benzaldehyd (AWC)	Butanol (AWC)
nuo-2	mild		↑	↑	
	strong	↓	↓	↓	↓
nuo-5	mild		↑		
	strong	↓	↓	↓	↓
F53F4.10	mild		↑		
	strong	↓	≈	↓	↓
nuo-1	mild				
	strong	↓			
lpd-5	mild		↑	↑	
	strong	↓	↓	≈	↓
T20H4.5	mild				
	strong	↓	↓	↓	

↑ Increased Chemotaxis index; ↓ decreased Chemotaxis index or ≈ no changes in the Chemotaxis index, compared to N2 worms fed bacteria expressing the empty-vector (pL4440)
Stage of the worms: L3 Larvae

Supplementary Table 4. Developmental rescue on *cep-1(lg12501)* mutants

C. elegans gene	Human gene	Rescuing strong phenotype with *cep-1*(lg12501)	Rescuing strong phenotype returning on Pl4440
lpd-5	NDUFS4	Partial rescue	Yes, returning on Pl4440 within 24 h
nuo-5	NDUFS1	Partial rescue	Yes, returning on Pl4440 as eggs
F53F4.10	NDUFV2	ND	Yes, returning on Pl4440 within 24 h
nuo-1	NDUFV1	Not rescued	Yes, returning on Pl4440 as eggs
T20H4.5	NDUFS8	Partial rescue	Yes, returning on Pl4440 as eggs
nuo-2	NDUFS3	Partial rescue	Yes, returning on Pl4440 within 24 h

ND not determined

Supplementary Table 5. Summary of results with lutein on *nuo-5* depleted animals

Phenotype		con	*nuo-5*	*nuo-5* + lutein
Development		wt	def	res
Pharyngeal pumping	Pump Frequency	wt	def	wt
	Pump Duration	wt	def	def
	IPI Duration	wt	def	wt
	R to E ratio	wt	def	def
Movement	In agar	wt	wt	ND
	In liquid	wt	def	wt
Sensory function	Attraction to Pyrazine	wt	def	wt
	Attraction to Butanol	wt	def	wt
Paralysis	Aldicarb	wt	def	wt
	Levamisole	wt	def	def
	PTZ - Head bobs	wt	wt	wt
	PTZ- Full body	wt	def	wt
ATP levels		wt	def	ND
mtDNA damage		wt	wt	ND
nDNA damage		wt	def	ND
Lipid/protein oxidation		wt	def	def
Resistance to oxidative stress		wt	def	def
ROS in elegans		wt	def	res
Gene's expression	*nuo-5*	wt	def	def
	hsp-6	wt	def	def
	gst-4	wt	def	def
	hlh-30	wt	def	wt
	dct-1	wt	def	res
	nlg-1	wt	def	wt

wt = wild type phenotype
def = defective
res = partial rescue
ND = not determined

Supplementary Table 6: Primers' list used for the qPCRs.

C. elegans gene	Forward primer (5'-3')	Reverse primer (5'-3')
vps-35	GGAATACGACCGACCAGGAG	TGCATCCATGGTTTTTCCCTTAT
nlp-40	GTTGCGGTTTTTGCGGCTC	ATCCAAGGAATGTGTCGGCT
unc-10	GGGGAGCAGAAGGGAAAAGA	CCAAGAGGACCATCAGCGAG
gbf-1	GGCTCGGAGACAATACCAACT	ATCGGCAACCTCATTCAGCA
F53B2.5	ATGCCATCACCAGTAGCTCG	AAGCATGTCGATCCGTGTGG
nuo-5	TCGAGCCGTTTCTGAGGTTT	GTGGGGAGCGATCTCATTCA
eif-3C	ACCACTCTGCAAACCGTCAA	ACTCTTGAGTGTCATCCTTCAG
csq-1	GCCAGGATCAGTTGCTCTCA	AAGTTGGGGCGAAAAGTGGA
**Mouse gene**	**Forward primer (5'-3')**	**Reverse primer (5'-3')**
mNLGN1	GGTACTTGGCTTCTTGAGCAC	AAACACAGTGATTCGCAAGGG
mNLGN2	TGTCATGCTCAGCGCAGTAG	GGTTTCAAGCCTATGTGCAGAT
mNLGN3	CCCTGGGCTTCCTCAGTTTG	GGCAATGGTACTCTGGCACC
mNLGN4-1	CCCAACGAAGATTGCCTCTAT [2]	TCCATCTTCCGTGGGCACATACAC
mNLGN4-2	CCCAACGAAGATTGCCTCTAT	CGTCATTATCCGCTAAGTCCTC
mNRXN1	AACGGACTGATGCTTCACACA	GATATTGTCACCTGACGCAGATT
mNRXN2	CGGCTCACCTGACGTTAAACA	TCCTTGCCTTTTGTCGGCTG
mNRXN3	GCACTATCCTACAGGCAACAC	GGCCGGTTATATTTGAAGGGGA
**Human gene**	**Forward primer (5'-3')**	**Reverse primer (5'-3')**
NLGN1	TGGCTGCACACTAAGCACCACG	GCAGTGTGGCAAGAGTGGGCAT
NLGN2	CGGTTTTCTCAGCACCGGGGAC	TTTTCACTGAGCCAGCGCAGGG

Supplementary Table 7: *C. elegans* strains used in this study

Name	Genotype	Source
N2	wild-type	CGC[a]
	p*glb-10*::GFP	[3]
SJ4100	zcIs13[p*hsp-6*::GFP]	CGC[a]
CL2166	dvIs19 [(pAF15)*gst-4p*::GFP::NLS] III	CGC[a]
JIN1679	jinEx10 [*hlh-30p*::hlh-30::GFP + *rol-6*(su1006)]	[4]
LX929	vsIs48 [*unc-17*::GFP].	CGC[a]
PE255	feIs5 [*sur-5p*::luciferase::GFP + *rol-6*(su1006)] X	[5-7], CGC[a]
IR1431	N2;Ex001[pdct-1DCT-1::GFP]	[8]
AU78	agIs219 [T24B8.5p::GFP::*unc-54* 3' UTR + *ttx-3p*::GFP::*unc-54* 3' UTR] III	[9, 10], CGC[a]
VC228	*nlg-1*(ok259) X	CGC[a]
TU3401	*sid-1*(pk3321) V	[11], CGC[a]
CL6114	*nre-1*(hd20) *lin-15b*(hd126)	[12]
	vjIs47[p*nlg-1*::GFP]IV	[13]
	vjIs105[p*nlg-1*::nlg-1-gfp]III	[13]
CRR100	*nlg-1*(ok259) X; crrEx4 [pD95.77; pDD04NeoR (Pmyo-2::GFP)]	[14]
CRR104	*nlg-1*(ok259) X; crrEx4 [pD95.77 (P*nlg-1*::*nlg-1*); pDD04NeoR (P*myo-2*::GFP)]	[14]
CB407	*unc-49*(e407) III	[15]

[a]CGC: *Caenorhabditis* Genetics Center (https://cgc.umn.edu/)

Supplementary Methods

Lifespan assay

Survival analysis started from hatching and was carried out at 20°C. Animals were scored as dead or alive and transferred every day on fresh plates during the fertile period, and then every other day or every 3 days until death. Worms were considered dead when they stop pharyngeal pumping and responding to touch. Worms that died because of internal bagging, desiccation due to crawling on the edge of the plates, or gonad extrusion were scored as censored. These animals were included in lifespan analyses up to the point of censorship and were weighted by half in the statistical analysis. We calculated mean lifespan, standard deviation of the mean, and P value (Mantel-Cox regression analysis) from Kaplan-Meyer survival curves of pooled population of animals coming from at least two independent replicas. For statistical analysis we used the Online Application for Survival analysis OASIS 2 [16].

Chemotaxis assays

Sodium azide (NaN_3) was used to anesthetize worms. It was placed on buffered agar 180 degrees opposite on a 10 cm dish; the attractant (or repellent) was then placed on one NaN3 spot, and ethanol (neutral odor for the worms in which chemical was diluted) on the other spot; a population of 80-100 age-synchronized animals was placed in the center of the testing plate and the number of worms at attractant and control was counted every 15 minutes for four hours to calculate the Chemotaxis Index (CI). CI=(A-B)/(A+B+C), where A is the number of worms at attractant, B is the number of worms at control and C is the number of animals which didn't reach any of the two spots at the end of the two hours. For a population of 3 days old, wild-type animals, a good CI is around 0.8 for attractants and –0.8 for repellents after two hours (CI=0 means no attraction, while CI=1 or –1 represent maximum attraction or repulsion, but there are always some animals that, also for a wild-type strain, remain randomly dispersed in the assay plate or reach the control spot instead of the attractant) [17].

Mitochondrial Respiration

Synchronized wild-type (N2) worms were grown at 20°C for two consecutive generations on bacteria expressing dsRNA against the genes of interest: *nuo-5* and *lpd-5*. Mitochondrial respiration from these animals was compared with stage-matched animals left untreated or fed for only one generation (mild treatment). The Seahorse Bioscience Flux Analyzer was programmed with the appropriate protocol [18]. Worms were rinsed off the plate and washed and the number of animals estimated (by counting the number of worms in 20µl drops under a dissecting microscope). The 24-well Seahorse Cartridge was loaded with nearly 250 nematodes pipetted into each well. The final volume of each well was brought to 525µl with unbuffered EPA water. Two wells per assay were filled with EPA water without worms and used as blanks. 75 µl of each drug were loaded into the designated injection ports of the seahorse cartridge. The final solution concentrations after injection of each drug being the following: 20µM DCCD (1% DMSO), 25µM FCCP (2% DMSO), and 10mM sodium azide. The OCR was normalized to the actual number of worms in each well and the values obtained were again normalized on the actual protein content per worms measured with a BCA protein assay after sonication of 2000 nematodes for each condition. The average and the standard error of OCR per worm across the five replicates for each sample were calculated and a minimum of 10 time points of 5 wells was used.

ATP levels

Worms at the desired developmental stage (L2/L3 larvae) were transferred to 15mL conical tube, spun at 2200 RCF and the supernatant was removed. Nematodes were resuspended in k-medium to a concentration of 1.0±0.2 nematodes/µl. 50-100 animals were pipetted into each well of a white 96-well plate (4-5 wells per treatment), and brought to a final volume 100uL with k-medium. K-medium alone was used for blank measurements. A plate reader was used to read first the GFP fluorescence (Emissions filter: 502nm; excitation filter: 485nm) and then the luminescence after injection of a luminescence buffer. The ATP production was then calculated dividing the luminescence signal by the GFP intensity.

Mitochondrial DNA copy number, mitochondrial DNA and nuclear DNA damage

Synchronized wild-type (N2) worms were grown at 20°C for two consecutive generations on bacteria expressing dsRNA against the genes of interest: *nuo-5 and, lpd-5.* nDNA and mtDNA from these animals was compared with stage-matched animals fed bacteria expressing the empty vector pL4440. Six worms were picked and pooled in a single tube per biological replicate, and three biological replicates were taken per treatment in three experiments separated in time.

This assay defines the control samples as undamaged and determines a lesion frequency in experimental samples based on any decrease in amplification efficiency relative to the control samples [19]. One nuclear genome target (9,3 kb) and one mitochondrial genome target (10,9 kb) were amplified. The DNA damage assay is able to quantitatively measure the number of polymerase-stalling lesions based on the amount of amplification obtained from the QPCR [20-22]. To calculate DNA copy number, we utilized a real-time PCR assay in which, by using a standard, the actual DNA content can be calculate [23, 24]. This method is particularly advantageous because the standard curve is run along with the samples and the actual number of copies can be calculated.

Quantification of Gene Expression through fluorescent transgene reporters

For the SJ4100 and CL2166 strains the GFP intensity of the total body was quantified (**Supplementary Fig. 4a**). Concerning the JIN1679 strain, for each animal the number of nuclei showing HLH-30 nuclear translocation (between the pharynx and the vulva) (**Supplementary Fig. 4b**) were counted with a macro specifically customized with ImageJ. With the KP3928 strain the intensity of the fluorescence was quantified in the pharynx, specifically, two circular area were designed with ImageJ corresponding to anterior and posterior pharyngeal bulbs and the GFP

intensity was quantified in the two areas (**Supplementary Fig. 11b**). For P_{glb-10}::GFP the intensity of the neuronal nuclei located in the pharyngeal area was quantified (**Supplementary Fig. 11c**). For the P_{dct-1}::GFP a circular region in the middle body was selected to quantify the GFP signal (**Supplementary Fig. 8g**). For the selection of the area and the quantification of the signal the software ImageJ (http://imagej.nih.gov/ij/) was used. For the LX929 the intensity of the second proximal head neuron was quantified (**Supplementary Fig. 11d**). With the vjIs47, vjIs105 and CRR104 strains the intensity of the fluorescence was quantified in the ventral nerve cord (VNC), specifically, a rectangular area was designed in the posterior half of the nematode, between the vulva and the tale, and the GFP intensity was quantified in the selected areas (**Fig. 7e and Fig. 8f**).

RNA extraction for Microarray

Worms at the desired larval stage (L3) were washed off three big agar plates spotted with the RNAi bacteria under study (*nuo-5*, *lpd-5* and pL4440 for control). The plates were supplemented either with 1 µM Lutein (Sigma Aldrich PHR1699) or 0.25 % DMSO for control. Individual *C. elegans* were collected into nuclease-free water and frozen at −80 °C until needed. Frozen worm pellets were re-suspended in lysis buffer and the same volume of stainless-steel beads (6.0 mm RNase free) was added. A TissueLyser II (Qiagen) was used with 2 shakes for 2 minutes at 30 Hz. Total RNA was prepared from each sample using the RNeasy Mini Kit and QIAshredder according to the manufacturer's instructions (Qiagen-74104 and 79654). Total RNA from 5 different biological replicas was extracted, on column purified to remove small RNA fractions, and treated with DNAse (RNeasy kit, Qiagen-74004).

Supplementary information references

1. Maglioni, S., et al., *Mitochondrial bioenergetic changes during development as an indicator of C. elegans health-span.* Aging (Albany NY), 2019. **11**(16): p. 6535-6554.
2. Zhang, B., et al., *Autism-associated neuroligin-4 mutation selectively impairs glycinergic synaptic transmission in mouse brainstem synapses.* J Exp Med, 2018. **215**(6): p. 1543-1553.
3. Schiavi, A., et al., *Iron-Starvation-Induced Mitophagy Mediates Lifespan Extension upon Mitochondrial Stress in C. elegans.* Curr Biol, 2015. **25**(14): p. 1810-22.
4. Lapierre, L.R., et al., *The TFEB orthologue HLH-30 regulates autophagy and modulates longevity in Caenorhabditis elegans.* Nat Commun, 2013. **4**: p. 2267.
5. Lagido, C., et al., *Rapid sublethal toxicity assessment using bioluminescent Caenorhabditis elegans, a novel whole-animal metabolic biosensor.* Toxicol Sci, 2009. **109**(1): p. 88-95.

6. Lagido, C., et al., *Bridging the phenotypic gap: real-time assessment of mitochondrial function and metabolism of the nematode Caenorhabditis elegans.* BMC Physiol, 2008. **8**: p. 7.
7. McLaggan, D., et al., *Impact of sublethal levels of environmental pollutants found in sewage sludge on a novel Caenorhabditis elegans model biosensor.* PLoS One, 2012. **7**(10): p. e46503.
8. Palikaras, K., E. Lionaki, and N. Tavernarakis, *Coordination of mitophagy and mitochondrial biogenesis during ageing in C. elegans.* Nature, 2015. **521**(7553): p. 525-8.
9. Shivers, R.P., et al., *Tissue-specific activities of an immune signaling module regulate physiological responses to pathogenic and nutritional bacteria in C. elegans.* Cell Host Microbe, 2009. **6**(4): p. 321-30.
10. Shivers, R.P., et al., *Phosphorylation of the conserved transcription factor ATF-7 by PMK-1 p38 MAPK regulates innate immunity in Caenorhabditis elegans.* PLoS Genet, 2010. **6**(4): p. e1000892.
11. Calixto, A., et al., *Enhanced neuronal RNAi in C. elegans using SID-1.* Nat Methods, 2010. **7**(7): p. 554-9.
12. Schmitz, C., P. Kinge, and H. Hutter, *Axon guidance genes identified in a large-scale RNAi screen using the RNAi-hypersensitive Caenorhabditis elegans strain nre-1(hd20) lin-15b(hd126).* Proc Natl Acad Sci U S A, 2007. **104**(3): p. 834-9.
13. Staab, T.A., et al., *Regulation of synaptic nlg-1/neuroligin abundance by the skn-1/Nrf stress response pathway protects against oxidative stress.* PLoS Genet, 2014. **10**(1): p. e1004100.
14. Calahorro, F. and M. Ruiz-Rubio, *Functional phenotypic rescue of Caenorhabditis elegans neuroligin-deficient mutants by the human and rat NLGN1 genes.* PLoS One, 2012. **7**(6): p. e39277.
15. Thapliyal, S. and K. Babu, *Pentylenetetrazole (PTZ)-induced Convulsion Assay to Determine GABAergic Defects in Caenorhabditis elegans.* Bio Protoc, 2018. **8**(17).
16. Han, S.K., et al., *OASIS 2: online application for survival analysis 2 with features for the analysis of maximal lifespan and healthspan in aging research.* Oncotarget, 2016.
17. Hart, A.C., *Behavior* in *WormBook, The C. elegans Research Community*. 2006.
18. Luz, A.L., et al., *Seahorse-based analysis of cellular respiration in Caenorhabditis elegans.* in preparation.
19. Meyer, J.N., *QPCR: a tool for analysis of mitochondrial and nuclear DNA damage in ecotoxicology.* Ecotoxicology, 2010. **19**(4): p. 804-11.
20. Furda, A.M., et al., *Analysis of DNA damage and repair in nuclear and mitochondrial DNA of animal cells using quantitative PCR.* Methods Mol Biol, 2012. **920**: p. 111-32.
21. Furda, A., et al., *Quantitative PCR-based measurement of nuclear and mitochondrial DNA damage and repair in mammalian cells.* Methods Mol Biol, 2014. **1105**: p. 419-37.
22. Hunter, S.E., et al., *The QPCR assay for analysis of mitochondrial DNA damage, repair, and relative copy number.* Methods, 2010. **51**(4): p. 444-51.
23. Rooney, J.P., et al., *PCR based determination of mitochondrial DNA copy number in multiple species.* Methods Mol Biol, 2015. **1241**: p. 23-38.
24. Venegas, V. and M.C. Halberg, *Measurement of mitochondrial DNA copy number.* Methods Mol Biol, 2012. **837**: p. 327-35.
