# Supplementary material for: Neuroligin-mediated neurodevelopmental defects are induced by mitochondrial dysfunction and prevented by lutein in C. elegans
Source: Nat Commun. 2022 May 12;13:2620. doi: 10.1038/s41467-022-29972-4 (PMC9098500; doi:10.1038/s41467-022-29972-4)
Supplement: Supplementary file 8 — Reporting Summary [file 41467_2022_29972_MOESM8_ESM.pdf]

nature portfolio

Corresponding author(s): Natascia Ventura

Last updated by author(s): Mar 11, 2022

Reporting Summary

Nature Portfolio wishes to improve the reproducibility of the work that we publish. This form provides structure for consistency and transparency in reporting. For further information on Nature Portfolio policies, see our Editorial Policies and the Editorial Policy Checklist.

Statistics

For all statistical analyses, confirm that the following items are present in the figure legend, table legend, main text, or Methods section.

n/a	Confirmed	
☐	☒	The exact sample size (*n*) for each experimental group/condition, given as a discrete number and unit of measurement
☐	☒	A statement on whether measurements were taken from distinct samples or whether the same sample was measured repeatedly
☐	☒	The statistical test(s) used AND whether they are one- or two-sided<br>*Only common tests should be described solely by name; describe more complex techniques in the Methods section.*
☐	☒	A description of all covariates tested
☐	☒	A description of any assumptions or corrections, such as tests of normality and adjustment for multiple comparisons
☐	☒	A full description of the statistical parameters including central tendency (e.g. means) or other basic estimates (e.g. regression coefficient) AND variation (e.g. standard deviation) or associated estimates of uncertainty (e.g. confidence intervals)
☐	☒	For null hypothesis testing, the test statistic (e.g. *F*, *t*, *r*) with confidence intervals, effect sizes, degrees of freedom and *P* value noted<br>*Give P values as exact values whenever suitable.*
☒	☐	For Bayesian analysis, information on the choice of priors and Markov chain Monte Carlo settings
☐	☒	For hierarchical and complex designs, identification of the appropriate level for tests and full reporting of outcomes
☒	☐	Estimates of effect sizes (e.g. Cohen's *d*, Pearson's *r*), indicating how they were calculated

Our web collection on statistics for biologists contains articles on many of the points above.

Software and code

Policy information about availability of computer code

Data collection	ZEN 2 (blue edition) software was used for taking images. Respiration data were acquired with Wave Desktop 2.3, the data acquisition software for the Seahorse XF24 Analyzer. For gene expression profile data were obtained using Affymetrix C. elegans Gene 2.0 ST Microarrays. The software NemAcquire 2.1 (https://invivobiosystems.com/product-category/instruments/screenchip-system-software/) were used for acquisition of pharyngeal pumping rate. BioRad iQ5.2 Software was used for collecting RT-PCR data.
Data analysis	Microsoft Excel (2019) and Graphpad Prism 8 software were used for data analysis. Image J (version 1.52h) was used for quantification of GFP signal with GFP expressing C. elegans strains. OASIS 2 was used for analysis of survival experiments (Han et al (2016) Oncotarget 11269).<br>For gene expression analysis the sofrtwares ClueGO v2.5, R (Bioconductor) and Partek Flow (build 7.0.18.1116) were used. R version 3.5.1 (2018-07-02), clusterProfiler_3.8.1, enrichplot_1.0.2, Bioconductor version 3.8, LIMMA version 3.36.5, oligo package 1.46.0 were used. The software NemAnalysis 2.0 (https://invivobiosystems.com/product-category/instruments/screenchip-system-software/) was used for analysis of pharyngel pumping rate. FTIR data was analysed using OPUS 7.5 (Brucker), Unscrambler 10.5 and Matlab R2010b (Mathworks) .

For manuscripts utilizing custom algorithms or software that are central to the research but not yet described in published literature, software must be made available to editors and reviewers. We strongly encourage code deposition in a community repository (e.g. GitHub). See the Nature Portfolio guidelines for submitting code & software for further information.

Data

Policy information about availability of data

All manuscripts must include a data availability statement. This statement should provide the following information, where applicable:

- Accession codes, unique identifiers, or web links for publicly available datasets
- A description of any restrictions on data availability
- For clinical datasets or third party data, please ensure that the statement adheres to our policy

The GEO accession numbers for microarray data reported in this paper are GSE144573 and GSE144574.
WormBase, OMIM and PubMed are publicly available databases.

Field-specific reporting

Please select the one below that is the best fit for your research. If you are not sure, read the appropriate sections before making your selection.

☒ Life sciences ☐ Behavioural & social sciences ☐ Ecological, evolutionary & environmental sciences

For a reference copy of the document with all sections, see nature.com/documents/nr-reporting-summary-flat.pdf

Life sciences study design

All studies must disclose on these points even when the disclosure is negative.

Sample size	Sample size was not predetermined using any statistical method. Nonetheless, in our study we followed standard protocols and procedures from the field. Thus, we used a sample size which is equal or above the average considered sufficient for each specific experimental setting.
Data exclusions	For the microarray data analysis although we collected five samples for each condition, one replicate (RII) was excluded from the analysis because very different from the other replicates and clustering close together.
Replication	Multiple trials/biological replicas of each experiment were conducted. Number of biological replicates are always indicated in the figure legends and were combined for data representation.
Randomization	Animals were randomly chosen for analysis. In each assay (lifespan, chemotaxis, paralysis.....) worms were randomly selected for analysis from larger population growth under every experimental condition.
Blinding	The main investigator is familiar with the C. elegans phenotypes upon mitochondrial dysfunction and could not carry out the experiments in blind. Therefore, to secure unbiased results, most of the assays were also carried out by different investigators (investigators were blinded to group allocation during data collection). However, the data analysis was carried out from the main investigator who must know how to group the samples for the analysis.

Reporting for specific materials, systems and methods

We require information from authors about some types of materials, experimental systems and methods used in many studies. Here, indicate whether each material, system or method listed is relevant to your study. If you are not sure if a list item applies to your research, read the appropriate section before selecting a response.

Materials & experimental systems

n/a	Involved in the study	
☒	☐	Antibodies
☐	☒	Eukaryotic cell lines
☒	☐	Palaeontology and archaeology
☐	☒	Animals and other organisms
☒	☐	Human research participants
☒	☐	Clinical data
☒	☐	Dual use research of concern

Methods

n/a	Involved in the study	
☒	☐	ChIP-seq
☒	☐	Flow cytometry
☒	☐	MRI-based neuroimaging

Eukaryotic cell lines

Policy information about cell lines

Cell line source(s)	Cell lines were obtained from patient-derived primary human skin fibroblasts. The use of patient-derived fibroblasts was approved by the local ethic committee of the Heinrich Heine University of Düsseldorf (study number #4272). Written informed consent was obtained from the parents.

Authentication	None of the cell lines used were authenticated.
Mycoplasma contamination	We confirm that all cell lines were tested negative for mycoplasma contamination.
Commonly misidentified lines (See ICLAC register)	No commonly misidentified cell lines were used in this study.

Animals and other organisms

Policy information about studies involving animals; ARRIVE guidelines recommended for reporting animal research

Laboratory animals	Organism: Caenorhabditis elegans<br>Sex: hermaphrodite<br>Strains: names and genotypes are listed in Supplementary Table 9.<br>Stage: experiments were performed using L3 stage animals except otherwise specified in the figure legends.
Wild animals	No wild animals were used in this study.
Field-collected samples	The study did not involve samples collected from the field.
Ethics oversight	Our research complies with all relevant ethical regulations. No ethical approvals are required for laboratory studies using the nematode Caenorhabditis elegans.

Note that full information on the approval of the study protocol must also be provided in the manuscript.
